# Physiological Action and Uses of Cocaine

**Published:** 1891-01

**Authors:** 


### SELECTIONS.

### Physiological Action and Uses of Cocaine.

The physiological action of cocaine will be understood most easily by contrast with that of curare. The latter is well known as the type of peripheral motor-nerve paralyzers; cocaine, on the contrary, exerts its action upon the peripheral sensory nerves. They resemble each other, according to Dastre, involving only the terminations of the nerves; the nerve trunks preserve their excitability, and may even have it exaggerated. Again, while curare paralyzes the vaso-constrictor nerves within the blood-vessels, cocaine, on the contrary, excites them.

Cocaine in small doses appears to act as a general stimulant, producing a feeling of exhilaration, with an increased flow of speech, wakefulness, and a more rapid development of ideas, without, however, increased capacity for brain work. In this respect cocaine resembles morphine by contrast with caffeine. The heart and respiration are accelerated. In larger doses it causes slight dimness of vision, a feeling of tention and constriction of the forehead, with perhaps slight headache, dizziness, and great restlessness. The latter symptom is due to hyper-excitability of the muscles, and is explained by the condition of the nerve trunks. In still larger doses the effect of cocaine in constricting the blood-vessels is evidenced by coldness of the extremities. The rise in blood-pressure which occurs is due in part to acceleration of the heart's action.

Respiration is accelerated by cocaine, while under poisonous doses it becomes embarrassed and shallow, and stops before the heart. The pupil is dilated through stimulation of the sympathetic system. General analgesia develops only late and under poisonous doses, so that it is of no clinical importance.

Toxic doses of cocaine produce, in some cases, death preceded by collapse, cyanosis, and convulsions, the respiration especially being affected. In some cases the patient becomes very restless and loquacious, the speech is thick, and the sentences unfinished, the mouth and throat dry, the patient is giddy and staggers on attempting to walk, and is covered with a cold, clammy sweat

In some cases collapse and unconsciousness have developed speedily, and have been succeeded by active delirium. Autopsies in fatal cases have demonstrated the presence of cerebral and visceral congestion, especially of the kidney.

Prolonged use of cocaine has resulted in chronic poisoning, and in the production of the "cocaine habit." The former is said to be characterized by great excitement, hallucinations chiefly of sight and hearing, and great weakness of the lower limbs almost amounting to paresis.

Cocaine is used in medicine and surgery principally as a local anæsthetic. The limits of its applicability have not yet been determined. It is most serviceable, however, when applied to the more delicate mucous membranes, such as those of the eye, nose, urethra, and vagina, where the peripheral sensory nerves are close to the surface, and from which absorption is rapid.

Space will not permit a detailed mention of all the cases in which cocaine can be used; in general it may be said to be wherever the irritated nerves can be reached, whether exposed in a hollow tooth, or bared by cancerous ulceration, as in the breast. Hypodermically it has been used with more or less success in the extraction of teeth, the opening of buboes, the excision of small tumors, to prevent the pain caused by iodine when thrown into the tunica vaginalis for the cure of hydrocele, and in various other minor surgical operations. It forms a useful addition to soothing ointments, such as those for acute eczema, or for pruritus.

Its property of contracting blood-vessels, and drying mucous membranes makes it especially serviceable in acute coryza. It is most effective in the congestive (first) stage, but even when there is a profuse watery or mucous discharge, it gives more relief than any other remedy. A watery solution should be used, and eight or ten drops of a four or five per cent. solution may be snuffed up each nostril as often as the necessities of the case demand.

Internally cocaine has been employed to check vomiting, and in some cases with great success. Gastralgia has also been relieved by it.

In what has just been said an effort has been made to point out the most important physiological actions of cocaine, and to suggest the classes of cases in which it is likely to prove useful, rather than to enumerate the separate affections in which it may be used. Whenever it is used, it should not be forgotten that cocaine is as much a poison as morphine or strychnine, and that, being more uncertain in its effects, it should be watched with more care. It would seem to be most dangerous to nervous patients with weak hearts.—*Med. & Surg. Reporter.*

